# Tumor Wide Horizontal Invasion Predicts Local Recurrence for Scrotal Extramammary Paget’s Disease

**DOI:** 10.1038/srep44933

**Published:** 2017-03-21

**Authors:** Lujia Wang, Chenchen Feng, Minwei Zhou, Zhongwen Zhou, Guanxiong Ding, Peng Gao, Qiang Ding, Zhong Wu

**Affiliations:** 1 Department of Urology, Huashan Hospital,Fudan University, 200040, Shanghai, PR China; 2 Department of General Surgery, Huashan Hospital,Fudan University, 200040, Shanghai, PR China; 3 Department of Pathology, Huashan Hospital,Fudan University, 200040, Shanghai, PR China

## Abstract

Extramammary Paget’s disease (EMPD) is a rare malignancy, and little was known about its prognostic factors and optimal treatment. In the current study, we aimed to discuss clinical and pathological features of scrotal EMPD and determine the prognostic factors for cancer-specific survival and local recurrence. A total of 206 patients with scrotal EMPD lesions surgically treated at our institute were studied. All clinical and pathological data were reviewed. Immunohistochemical staining of TP53 and Ki67 was examined as well. At the last follow-up, 175 patients (84.95%) were alive. Twelve patients (5.83%) had died of the disease due to distant metastases. Fifteen patients (7.28%) developed local recurrences of scrotal EMPD. Ki67 expression was significantly elevated in patients with wide horizontal invasion (P = 0.003). In univariate analysis, high invasion level, presence of nodule, presence of lymphovascular invasion, adnexa invasion, lymph node metastasis and high p53 expression were significant factors for poor cancer-specific survival. In multivariate analysis, high p53 expression was significantly correlated with poor cancer-specific survival. Wide horizontal invasion was independently correlated with local recurrence-free survival of scrotal EMPD. In conclusion, wide horizontal invasion is an independent risk factor for local recurrence-free survival in the patients with scrotal EMPD.

Extramammary Paget’s disease (EMPD) is a rare malignancy that mainly affects the anogenital region in elderly people. In men, the scrotum is more often involved than the penis, and the disease is usually misdiagnosed as eczema. It is considered to be most likely derived from the undifferentiated pluripotent cells of the epidermis^1^. The disease can be classified as primary or secondary EMPD. Primary EMPD arises as an *in situ* tumor in the epidermis, while secondary EMPD involves direct expansion to the skin from underlying neoplasm, commonly a rectal or genitourinary carcinoma^2^.

Most patients with primary EMPD have a good prognosis, because the tumor cells grow slowly and the lesion is usually limited to the epidermis^3^. However, in some cases the tumor can present aggressive behavior and invades the dermis and subcutaneous tissue. Once the tumor invades the dermis, the risk of lymph node metastasis increases, and could result in a poor prognosis^4^,^5^. With regard to treatment, complete surgical excision is the first choice for patients with primary EMPD without distant metastasis and a complete cure can be expected in most cases^6^,^7^. On the contrary, the treatment effect for invasive EMPD with metastasis is often disappointed as no standardized highly effective therapy has been developed presently^8^.

The method of surgical excision and defining the surgical margin of EMPD remain controversial. At present, surgical modalities including Mohs micrographic surgery, fluorescent dyes and frozen section examination (FSE) are recommended to ensure clear margins. However, even extensive resections are complicated by a high local recurrence rate due to several characteristics including tumor multifocality and ill-defined margins^9^,^10^.

Due to the rarity of scrotal EMPD, little was known about its prognostic factors and optimal treatment. In this study, we aimed to discuss a few clinical and pathological features of the scrotal EMPD and determine the prognostic factors for cancer-specific survival and local recurrence.

## Material and Methods

### General information

A total of 206 patients with scrotal EMPD lesions were included in this study. All patients were surgically treated between April 2003 and May 2015 at the department of Urology of Huashan Hospital. All lesions were primary EMPD and the cases of secondary EMPD were excluded.

### Treatment of scrotal EMPD and patient follow-up

All patients were primarily diagnosed by biopsy. Complete physical examination, ultrasonography, and pelvic computed tomography (CT) were performed preoperatively to identify potential local or distal lymph node involvement. Wide surgical excision of skin lesions was performed in all patients of EMPD. Surgical resection margins were assessed to be negative in all cases. In patients with suspected local invasion, the deepest cut reached deep fascia. Surgical excision was performed initially approximately 2.0 cm from the visualized margin of the lesion. Intraoperative frozen section examination (FSE) was performed immediately after the lesion was completely removed. FSE was performed using the bread-loafing method. Excision was widened for another 1 cm on the margin-positive side if FSE was positive until a negative margin was acquired. All patients who were proved to have metastasis in lymph nodes typically underwent subsequent therapeutic lymph node dissection. Closure was customized to the size of the lesion and included scrotal skin release angioplasty, pedicle flap repair, and skin grafting. As for follow-up, patients were monitored for local recurrence, underlying pelvic malignancies and systemic metastasis by physical examination every 3 to 6 months and imagine tests, including chest X-ray, CT or ultrasonography. Patients who had experienced local recurrence underwent surgical excision again.

The clinical and demographic data on all patients were recorded and analyzed preoperatively, that included age, delay in diagnosis, tumor size, multiplicity, recurrent disease, presence of nodules, and presence of ulceration. Delay in diagnosis was defined as the time from onset of symptoms until diagnosis. Large tumor size was defined as visualized tumor area >25.0 cm^2^. Pathological data were acquired postoperatively, including invasion level, adnexa invasion, lymphovascular invasion, horizontal invasion and lymph node metastasis. Since there is no specific tumor-node-metastasis (TNM) classification accepted worldwidely in EMPD, invasion level could be stratified by three groups in accordance with previous studies^11^, including *in situ* in the epidermis (IE), microinvasion into the papillary dermis (MI) and deep invasion into the reticular dermis (DI). For heterogeneity conditions, lesions that involved different invasion levels were classified according to the deepest site of invasion identified in the specimen. Adnexa invasion was defined as tumor invading cutaneous adnexa including sweat glands and folliculi pili. Wide horizontal invasion was defined as tumor invaded laterally beyond the visualized margin >2.0 cm.

Written informed consent was obtained from each patient before any study-specific investigation was performed. This study was carried out in accordance with the ethical standards of Helsinki Declaration II and approved by the Institution Review Board of Huashan Hospital, Fudan University.

### Histology and Immunohistochemistry

Resection specimens were fixed in 10% formalin and embedded in paraffin, and histologic sections were obtained and stained with hematoxylin and eosin (H&E) by routine methods. All sections were reviewed independently by two pathologists without knowledge of patient profile. Before immunohistochemistry, heat-mediated antigen retrieval was performed by boiling the slides in 0.01 M citrate buffer at pH6.0 for 20 min in a microwave oven. The primary antibodies were diluted, including p53 (clone1B10, Novocastra, Newcastle, UK) at 1:50 and Ki67 (clone MM1, Novocastra, Newcastle, UK) at 1:100. The slides were stained immunohistochemically using the avidin-biotin-complex method for both antibodies. Finally, the slides were dehydrated through graded alcohols to xylene and mounted in a mounting medium. For positive controls, colon caricinoma was used for Ki67 and p53. For negative controls, all primary antibodies were omitted.

Staining and scoring protocols for p53 and Ki67 were previously described^12^,^13^. Ki67 was scored by label index while p53 value was scored semi-quantitatively as 0 for negative, 1 for mild, 2 for moderate, and 3 for strong. The Ki67 labeling index was considered high when samples demonstrated 20% or greater positivity. The expression of p53 was considered of when samples were scored 2 to 3. All experimental protocols were approved by the Huashan institutional review board of Fudan University.

### Statistics

The SPSS 17.0 for Windows program was used for statistical analysis. All data were presented as mean ± standard deviation (SD). The Student’s t-test was applied to compare scores of Ki67 and p53 between two groups, while analysis of variance (ANOVA) was used for comparisons in more than 2 groups. Categoric variables were analyzed using the Chi-square test or Fisher exact test where applicable. Kaplan-Meier method was used to evaluate cancer-specific survival and local recurrence-free survival while survival curves were compared using the Log-rank test. Multivariate Cox proportional regression analysis was performed to determine the independent contribution of clinicopathological factors to cancer-specific survival and local recurrence-free survival. The end-point variables of interest were cancer specific death and local tumor recurrences, respectively. A p value of <0.05 was considered statistically significant.

## Results

### Population characteristics

Demographics and clinical data are given in Table 1. All the patients were male Chinese, with the age at diagnosis ranging from 45 to 91 years (mean, 67.23 years). During the operation, lymph node or sentinel lymph node biopsy was performed on 21 cases and 10 (4.85%) of them underwent subsequent lymph node dissection because of lymph node metastasis. Radiation therapy was performed on 4 patients (1.94%) and systemic chemotherapy on 9 patients (4.37%) due to distant metastases that occurred postoperatively during follow-up.

### Pathological characteristics

**Figure 1:**
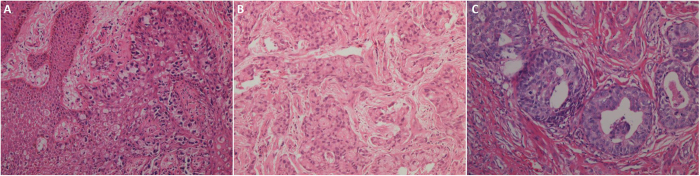
Histopathology of scrotal EMPD. (**A**) Within the epidermis; (**B**) Invades the dermis; (**C**) Adnexa invasion. (H&E, capture at ×200).

**Figure 2:**
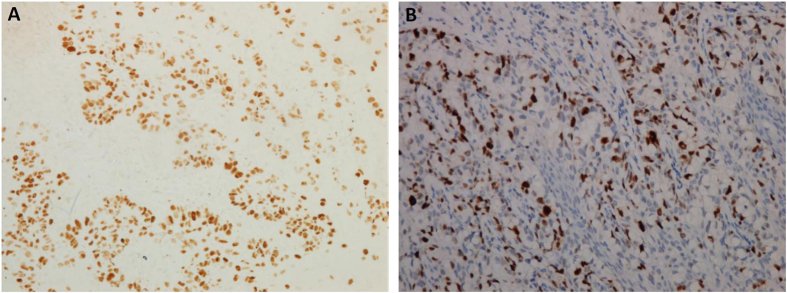
Immunohistochemistry of (**A**) p53 and (**B**) Ki67 in scrotal EMPD. (capture at ×200).

According to the deepest site of tumor invasion, IE, MI and DI were involved in 183, 10 and 13 cases, respectively (Fig. 1A,B). Lymphovascular invasion was found in 8 cases, whereas adnexa invasion was noted in 27 cases (Fig. 1C). Fifty-two patients had wide horizontal invasion. As for immunohistochemical analyses (Fig. 2A,B), moderate and strong p53 expressions were observed in 8 cases (3.88%). As for Ki67 expression, the mean Ki67 labeling index was 33.38 ± 20.29% (range, 0–90%), and high Ki67 expression was observed in 132 cases (64.08%).

### Immunohistochemical analyses

Expression levels of Ki67 and p53 are summarized in Table 2. Ki67 expression was significantly elevated in patients with wide horizontal invasion (P = 0.003). Significantly high rate of p53-positive expression was observed in the patients with lymphovascular invasion (P = 0.028) and lymph node metastasis (P = 0.040). Among p53-positive patients, the ulcerative lesions were correlated with significantly high level of p53 expression (P = 0.028).

### Cancer-specific survival

The median follow-up period was 30.5 months (range, 2–139). At the last follow up, 175 patients (84.95%) were alive, one of them had experienced bilateral inguinal lymph node metastasis. Twelve patients (5.83%) had died of the scrotal EMPD due to distant metastases, such as in bone, lung, liver, and/or multiple lymph node regions, and 7 patients had died of other causes. Table 3 shows the results of univariate analysis of factors affecting cancer-specific survival. High invasion level (P < 0.0001), nodule formation (P < 0.0001), presence of lymphovascular invasion (P < 0.0001), adnexa invasion (P = 0.017), lymph node metastasis (P < 0.0001) and high p53 expression (P = 0.026) were significant prognostic factors for poor cancer-specific survival. However, in the multivariate analysis, high expression of p53 was significantly correlated with poor cancer-specific survival (high expression *vs* low expression, HR 152.28; 95CI% 1.665–1.393E4; P = 0.029) (Table 4).

### Local recurrence-free survival

As of October 2015, 15 patients (7.28%) developed local recurrences of scrotal EMPD and 159 patients (77.18%) were alive without local recurrence. The univariate analyses showed no significant correlation between local recurrence-free survival and any of the clinicopathological parameters. However, the multivariate Cox proportional regression analysis identified that wide horizontal invasion (HR 5.142, 95%CI 1.262–20.956) was a significant risk factors for the local recurrence of scrotal EMPD (P = 0.022) (Table 5).

## Discussion

Up till now, few studies of large numbers of patients with genitalia EMPD have been reported, probably due to its rarity. This study included 206 patients with the scrotal EMPD over a 12 year period in a single institution, which is a very large cohort so far. That is mainly attributed to the high-profile dermatology department at our hospital. The department of dermatology of Huashan Hospital is the biggest dermatology center in China and its annually average outpatient visits were over 1.5 million in recent years, which has gathered a large number of patients with complicated or rare skin diseases throughout the country.

Increasing the depth of invasion has consistently been found to decrease the cancer-specific survival. Hatta *et al*.^11^ reported that the invasion level (IE, MI or DI) was the most significant factor associated with decreased survival by the multivariate analysis. Deeply invasive EMPD has been clearly shown to portend a poorer prognosis than noninvasive diseases^14^,^15^. Dai *et al*.^15^ stated that depth of invasion was an independent prognostic factor in the invasive EMPD, and the invasion of lower dermis (reticular layer) or deeper had significantly shorter cancer-specific survival. However, the clinical significance of MI has been controversial. MI was defined as the presentation of Paget cells to a depth of less than 1mm below the basement membrane previously^16^. In a study on the vulvar EMPD by Crawford *et al*.^17^, only deep invasion portended a worse prognosis, whereas minimally invasive disease did not. Shiomi *et al*.^18^ reported that nodal metastasis was more frequent in cases with the dermal invasion >1 mm, and they regarded it as a useful marker for prognosis of the penoscrotal EMPD. In our series, invasion level showed significant correlation with cancer-specific survival in the univariate analysis but failed to be proved as an independent prognostic factor in the multivariate analysis.

Several clinical studies have reported that regional lymph node metastasis is associated with worse outcomes than localized diseases in EMPD^6^,^11^,^19^. Moreover, one study has suggested that ipsilateral inguinal lymph node metastasis has better outcomes than bilateral inguinal lymph node metastasis^15^. In our study, lymph node metastasis was associated with poor cancer-specific survival in univariate analysis. Due to the small number of patients with inguinal lymph node metastasis, we were not able to stratify patients into different groups according to the different regions of positive inguinal lymph node.

The diagnosis of EMPD is often delayed because the common initial symptoms of pruritus and skin erythematous are relatively nonspecific. Patients often give a history of pro-longed treatment, such as topical corticosteroid or antifungal agents before diagnosis. In early lesion, scrotal EMPD could be clinically confused with the diseases such as eczematous dermatitis, psoriasis and leukoderma. Over time, the EMPD lesions may become erosive, ulcerated and scaly. At a later stage, the infiltrated lesions, even nodules may develop^20^.

In our series, the mean time from the awareness of skin lesions to diagnosis was 3.42 years (range, 0.25–20 years). In the study of Shu *et al*., univariate analysis, delay in diagnosis more than 7.5 years had a significantly shorter cancer-specific survival in invasive penoscrotum EMPD^21^. In our study, however, delay in diagnosis of scrotal EMPD more than 7 years was neither correlated with cancer-specific survival nor local recurrence-free survival.

With regard to histopathological features, the presence of nodules in the primary lesion was reported to be associated with shorter survival time^11^,^22^, which was in accordance with our results. Although the presence of nodules does not always represent dermal invasion diseases, we also believe it is clinically important. Moreover, in our study, the presence of ulceration of the lesion did not correlate with poor outcomes in univariate analysis.

Other histopathological features of the EMPD lesion, including lymphovascular invasion and adnexa invasion, were proved to be correlate with short cancer-specific survival in the univariate analysis. However, results from the multivariate analysis revealed that none of them were associated with cancer-specific survival. These results were consistent with the previous reports^15^,^21^. Some researchers believe that lymphovascular invasion is an independent predictor for the occult metastasis in clinically node-negative patients, since histological existence of lymphovascular invasion is often recognized far before the clinically visible lymph node metastasis occurs^15^. Choi *et al*.^23^ reported that lymphovascular invasion was an independent prognostic factor for predicting late recurrence of EMPD in multivariate analysis. However, in our cohort, no significant correlation was observed between lymphovascular invasion and local recurrence-free survival. Moreover, a histological study of EMPD reported that the degree of adnexal involvement was not associated with tumor progression^24^.

Some studies demonstrated that clear margins were highly relevant to whether local recurrence occurs^20^. Therefore major techniques including Mohs micrographic surgery, fluorescent dyes and frozen biopsy were extensively applied to defining the actual margin during surgery^25^. Although these methods could eventually ensure the clear margins, local recurrence persisted. Lee *et al*.^26^ reported that the local recurrence rate was 10% for Mohs micrographic surgery versus 30% for frozen section plus wide excision and the most of the recurrence cases exhibited negative margins.

In our series, surgical resection margins were pathologically confirmed negative in all cases. Our results indicated that wide horizontal invasion was independently correlated with local recurrence-free survival in multivariate analysis. The occurrence of the wide horizontal invasion indicated an unpredictable degree of subclinical tumor extension and a higher potential of tumor lateral invasion. Therefore, the regular follow-up is particularly essential for those patients.

Ki67 is a protein expressed in all active parts of the cell cycle and is an indicator of cell proliferation and a measure of cell growth fraction. Its expression is often quantified according to the percentage of immunopositive cells rather than grading category^27^. A previous study of Ki67 expression in EMPD showed no difference in Ki67 expression that identified cases with and without invasive disease, and therefore Ki67 expression probably cannot be used as a prognostic parameter for EMPD lesions^28^. Our previous study found that Ki67 expression alone is not associate with margin status, and its expression was not related to local recurrence^1^. In the present study, although high Ki67 expression level was neither correlated with cancer-specific survival nor local recurrence-free survival, it was proved to be associated with the wide horizontal invasion of EMPD, which could indirectly demonstrate the predictive value of Ki67 on tumor recurrence.

TP53 is the most frequently detected genetic abnormalities of tumor suppressor genes among human malignancies. It prevents cells from passing the G1 cell-cycle boundary, and negatively regulates cellular proliferation in response to genotoxic stress. This inhibition provides the cell time to correct DNA damage before mitosis, thereby preventing the passing of damaged DNA to daughter cells. Mutation of TP53 may result in uncontrolled cellular proliferation, accumulation of DNA damage and ultimately to cancer^29^. However, very few studies reported the prognostic value of p53 in scrotal EMPD. It was reported that the p53 status did not demonstrate any association with the invasive properties of EMPD^29^,^30^. In our study, high p53-negative rate was unexpectedly associated with lymphovascular invasion and lymph node metastasis, both of which were worse prognostic factors of EMPD. Negative p53 expression could be regarded as unmutated TP53 gene. We speculated that some unidentified mechanisms could be associated with those factors in p53-negative cases, and further studies would be warranted. Moreover, our results demonstrated that, in p53 positive cases, high p53 expression was significantly correlated with cancer-specific survival. Although the cases that died of EMPD are relatively small in the present study, which made it difficult to support that p53 independently associated with poor prognosis, we could conclude that p53 was an important prognostic marker for the scrotal EMPD.

## Conclusion

In conclusion, the wide horizontal invasion is an independent risk factor for local recurrence-free survival in the patients with scrotal EMPD. Moreover, in the p53-positive cases, high p53 expression is an important prognostic factor for scrotal EMPD.

## Additional Information

**How to cite this article**: Wang, L. *et al*. Tumor Wide Horizontal Invasion Predicts Local Recurrence for Scrotal Extramammary Paget’s Disease. *Sci. Rep.* **7**, 44933; doi: 10.1038/srep44933 (2017).

**Publisher's note:** Springer Nature remains neutral with regard to jurisdictional claims in published maps and institutional affiliations.

## Figures and Tables

**Table 1:** Demographics and clinical data.

Parameter		n (%)
Age (years)		67.23 ± 8.970^a^
Delay in diagnosis (years)		3.42 ± 3.135
Recurrent disease		23 (11.17%)
Large tumor size (>25.0 cm^2^)		86 (41.75%)
Multiple lesions		23 (11.17%)
Invasion level	*In situ*	183 (88.84%)
	Micro-invasion	10 (4.85%)
	Deep invasion	13 (6.31%)
Nodule formation		9 (4.37%)
Ulceration		10 (4.85%)
Lymphovascular invasion		8 (3.88%)
Adnexa invasion		27 (13.11%)
Wide horizontal invasion		52 (25.24%)
Lymph node metastasis		10 (4.85%)
**Total**		206

^a^All numerical variables were presented as mean ± standard deviation.

**Table 2:** Ki67 and p53 expressions in relation to clinicopathological parameters (mean ± SD).

		Ki67(%)^b^	p53	
			Positive expression (n,%)^c^	Expression score^b,d^
Delay in diagnosis (years)	<7	33.45 ± 20.18	116 (63.4%)	1.04 ± 0.31
	≥7	32.96 ± 21.46	15 (65.2%)	1.18 ± 0.41
	P	0.915	0.864	0.162
Tumor size (cm^2^)	≤25.0	34.98 ± 20.65	69 (57.5%)	1.04 ± 0.26
	>25.0	31.33 ± 19.79	60 (69.8%)	1.06 ± 0.39
	P	0.255	0.073	0.806
Multiple lesions	Absent	34.01 ± 20.35	114 (62.3%)	1.06 ± 0.35
	Present	29.05 ± 19.85	16 (69.6%)	1.00 ± 0.00
	P	0.296	0.496	0.056
Invasion level	*In situ*	33.80 ± 20.41	117 (63.9%)	1.04 ± 0.33
	Micro-invasion	23.56 ± 20.88	7 (70.0%)	1.17 ± 0.41
	Deep invasion	35.91 ± 17.72	6 (46.2%)	1.00 ± 0.00
	P	0.312	0.406	0.643
Nodule formation	Absent	33.21 ± 19.95	126 (64.0%)	1.05 ± 0.33
	Present	37.14 ± 28.70	4 (44.4%)	1.00 ± 0.00
	P	0.732	0.295	0.836
Ulceration	Absent	32.99 ± 19.77	123 (62.8%)	1.03 ± 0.27
	Present	39.40 ± 27.87	7 (70.0%)	1.33 ± 0.82
	P	0.334	0.643	0.028*
Lymphovascular Invasion	Absent	33.27 ± 20.28	129 (65.2%)	1.05 ± 0.33
	Present	35.50 ± 21.82	2 (25.0%)	1.00 ± 0.00
	P	0.763	0.028*	0.836
Adnexa invasion	Absent	32.55 ± 20.16	111 (62.0%)	1.04 ± 0.34
	Present	37.59 ± 20.86	19 (70.4%)	1.08 ± 0.29
	P	0.239	0.401	0.691
Wide horizontal	≤2.0	32.07 ± 19.18	95 (61.7%)	1.05 ± 0.37
	>2.0	44.49 ± 23.67	35 (67.3%)	1.04 ± 1.92
	P	0.003**	0.468	0.840
Lymph node metastasis	Absent	33.77 ± 20.24	127 (64.8%)	1.05 ± 0.33
	Present	26.56 ± 21.20	3 (30.0%)	1.00 ± 0.00
	P	0.301	0.040*	0.884

**Correlation is significant at the 0.01 level (2-tailed). *Correlation is significant at the 0.05 level (2-tailed).

^b^The student *t* test was applied to compare scores of ki67 and p53 between two groups. Analysis of variance (ANOVA) was applied for comparison of more than 2 groups.

^c^*Chi*-square analysis was applied for comparison of p53-positive rate between different groups. Fisher exact tests were used if any expected value was less than 5.

^d^All p53-negative patients were excluded.

**Table 3:** Univariate analysis for cancer-specific survival.

Parameter		Patients(n)	Died of disease(n)	P^e^
Age(years)	<70	184	11	0.530
	≥70	22	1	
Delay in diagnosis(years)	<7 years	183	10	0.651
	≥7 years	23	2	
Tumor size(cm^2^)	≤25.0	120	8	0.482
	>25.0	86	4	
Multiple lesions	Absent	183	12	0.169
	Present	23	0	
Invasion level	*In situ*	183	6	<0.0001**
	Micro-invasion	10	0	
	Deep invasion	13	6	
Nodule formation	Absent	197	9	<0.0001**
	Present	9	3	
Ulceration	Absent	196	12	0.404
	Present	10	0	
Lymphovascular invasion	Absent	198	5	<0.0001**
	Present	8	7	
Adnexa invasion	Absent	178	8	0.017*
	Present	28	4	
Wide horizontal invasion	≤2.0 cm	154	10	0.584
	>2.0 cm	52	2	
Recurrence disease	Absent	183	10	0.651
	Present	23	2	
Lymph node metastasis	Absent	196	5	<0.0001**
	Present	10	7	
Ki67 expression	<20% positive	68	5	0.836
	≥20% positive	125	8	
p53 expression	Negative	72	9	0.026*
	Low expression	115	1	
	High expression	8	1	

**Correlation is significant at the 0.01 level (2-tailed). *Correlation is significant at the 0.05 level (2-tailed).

^e^Survival curves were compared using the Log-rank test.

**Table 4:** Multivariate Cox regression analysis for cancer-specific survival.

Variables		HR	95%CI	P^f^
Age	(<70 years/≥70years)	0.001	0.000–3.227E25	0.844
Delay in diagnosis	(<7years/≥7years)	0.000	0.000–1.182E55	0.900
Recurrence disease	(Absent/Present)	0.002	0.000–4.094E47	0.915
Tumor size	(≤25 cm^2^/>25 cm^2^)	1.196	0.054–26.693	0.910
Multiple lesions	(Absent/Present)	0.001	0.000–3.142E48	0.904
Nodule formation	(Absent/Present)	0.161	0.000–2.942E54	0.978
Lymphovascular invasion	(Absent/Present)	1.397E16	0.000–1.21E111	0.739
Adnexa invasion	(Absent/Present)	0.000	0.000–2.219E18	0.686
Wide horizontal invasion	(≤2.0 cm/>2.0 cm)	1.191	0.034–41.951	0.923
Invasion level		—	—	0.473
	*In situ*	1	—	—
	Micro-invasion	0.001	0.000–2.945E65	0.933
	Deep invasion	10.742	0.237–486.051	0.222
Lymph node metastasis	(Absent/Present)	0.000	0.000–2.463E75	0.807
p53 expression		—	—	0.045*
	Negative	1	—	—
	Low expression	0.591	0.033–10.508	0.720
	High expression	152.280	1.665–1.393E4	0.029
Ki67 expression	(<20%/≥20% positive)	0.554	0.021–14.446	0.723

**Correlation is significant at the 0.01 level (2-tailed). *Correlation is significant at the 0.05 level (2-tailed).

^f^Multivariate Cox proportional regression was performed.

**Table 5:** Multivariate Cox regression analysis for local recurrence-free survival.

Variables		HR	95%CI	P^f^
Age	(<70 years/≥70 years)	0.256	0.028–2.347	0.228
Delay in diagnosis	(<7 years/≥7 years)	0.000	0.000–3.91E305	0.973
Recurrence disease	(Absent/Present)	0.594	0.066–5.340	0.642
Tumor size	(≤25 cm^2^/>25 cm^2^)	0.514	0.137–1.938	0.326
Multiple lesions	(Absent/Present)	0.924	0.219–3.903	0.914
Nodule formation	(Absent/Present)	0.000	—	0.986
Lymphovascular invasion	(Absent/Present)	0.001	—	0.997
Adnexa invasion	(Absent/Present)	0.000	0.000–8.91E289	0.971
Wide horizontal invasion	(≤2.0 cm/>2.0 cm)	5.142	1.262–20.956	0.022*
Invasion level		—	—	0.999
	*In situ*	1	—	—
	Micro-invasion	0.000	—	0.976
	Deep invasion	0.000	—	0.983
Lymph node metastasis	(Absent/Present)	2.347	—	1.000
p53 expression		—	—	0.418
	Negative	1	—	—
	Low expression	4.248	0.496–36.370	0.187
	High expression	0.000	—	0.982
Ki67 expression	(<20%/≥20% positive)	1.566	0.369–6.648	0.543

**Correlation is significant at the 0.01 level (2-tailed). *Correlation is significant at the 0.05 level (2-tailed).

^f^Multivariate Cox proportional regression was performed.
